# Reduction of Central Line-associated Bloodstream Infection Through Focus on the Mesosystem: Standardization, Data, and Accountability

**DOI:** 10.1097/pq9.0000000000000272

**Published:** 2020-03-25

**Authors:** Roshni Mathew, Alison Simms, Matthew Wood, Kristine Taylor, Sarah Ferrari, Michelle Rhein, Dionne Margallo, Lisa C. Bain, Amy K. Valencia, Jessey Bargmann-Losche, Lane F. Donnelly, Grace M. Lee

**Affiliations:** From the * Department of Pediatrics, Stanford University School of Medicine, Palo Alto, Calif.; † Center for Pediatric and Maternal Health, Stanford Children’s Health, Palo Alto, Calif.; ‡ Center for Professional Excellence and Inquiry, Stanford Children’s Health, Palo Alto, Calif.; § Neonatal Intensive Care Unit, Lucile Packard Children’s Hospital, Palo Alto, Calif.; ¶ Department of Radiology, Stanford University School of Medicine, Palo Alto, Calif.

## Abstract

**Introduction:**

Efforts to reduce central line-associated bloodstream infection (CLABSI) rates require strong microsystems for success. However, variation in practices across units leads to challenges in ensuring accountability. We redesigned the organization’s mesosystem to provide oversight and alignment of microsystem efforts and ensure accountability in the context of the macrosystem. We implemented an A3 framework to achieve reductions in CLABSI through adherence to known evidence-based bundles.

**Methods:**

We conducted this CLABSI reduction improvement initiative at a 395-bed freestanding, academic, university-affiliated children’s hospital. A mesosystem-focused A3 emphasized bundle adherence through 3 key drivers (1) practice standardization, (2) data transparency, and (3) accountability. We evaluated the impact of this intervention on CLABSI rates during the pre-intervention (01/15-09/17) and post-intervention (07/18–06/19) periods using a Poisson model controlling for baseline trends.

**Results:**

Our quarterly CLABSI rates during the pre-intervention period ranged from 1.0 to 2.3 CLABSIs per 1,000 central line-days. With the mesosystem in place, CLABSI rates ranged from 0.4 to 0.7 per 1,000 central line days during the post-intervention period. Adjusting for secular trends, we observed a statistically significant decrease in the post versus pre-intervention CLABSI rate of 71%.

**Conclusion:**

Our hospital-wide CLABSI rate declined for the first time in many years after the redesign of the mesosystem and a focus on practice standardization, data transparency, and accountability. Our approach highlights the importance of alignment across unit-level microsystems to ensure high-fidelity implementation of practice standards throughout the healthcare-delivery system.

## Abstract 2

Supplemental Digital Content is available in the text.

## INTRODUCTION

Central line-associated bloodstream infections (CLABSI) are a major healthcare-associated infection (HAI).^1^ CLABSI is associated with both increased morbidity and duration of hospitalization, resulting in a significant increase in hospital costs.^2^ The rate of CLABSI has been declining in the United States, with standardized infection ratios (SIR) for acute care hospitals decreasing by 19% between 2016 and 2017 compared with national baseline.^3^ Despite these declines, the estimated total direct medical costs due to CLABSIs is between $0.7 and $2.7 billion annually.^4^ Adherence to the central line insertion and maintenance bundles, which are evidence-based practices performed together, has been shown to reduce the rates of CLABSIs.^5–8^ However, having a hospital policy for a central line bundle or having moderate adherence to the bundle is insufficient for the reduction of CLABSI. An adherence of 95% or greater is associated with a decline in CLABSI.^8^ Despite knowing what to do, implementation remains challenging.

Healthcare systems commonly rely on clinical microsystems—an interdependent group of healthcare providers who work together regularly to provide direct care typically at a unit level—for implementation of hospital initiatives.^9,10^ Clinical microsystems are known to be important to optimize quality, safety, and overall care delivery.^11,12^ Despite having a robust microsystem infrastructure in our healthcare system, we encountered challenges that can mitigate the strengths of a microsystem approach. First, the reliance on microsystems to define optimal processes for various populations results in variability from clinical unit to unit in whether and how standards are modified. Second, as microsystems adopt and iterate on variations of the bundle, we moved away from adherence to *all* of the bundle elements and focused instead on performance on individual elements to define success. This focus led to a perception that despite following most of the bundle elements, CLABSI rates were immutable. Third, microsystems also adapt performance measures specific to each unit, which meant there was an emphasis on different measures in each unit. As an example, due to gestational age restrictions on the use of chlorhexidine in neonates, the neonatal intensive care unit (NICU) developed an alternate bathing protocol for their patients. These differences led to migration away from common standards, limiting transparency/comparability, and making accountability challenging for an entire system. This variation also led to confusion for providers, caregivers, and patients when they moved between clinical units.

We recognize that we cannot achieve meaningful and sustained improvements without successful microsystems. However, microsystems working in silos without a well-developed mesosystem can be an important gap.^11,13^ The mesosystem is the link between the microsystems and macrosystem levels. It is the group that sets expectations and performance measures, identifies barriers, provides evidence-based standards, and links strategy, operations, and the different microsystems.^12^ As part of our CLABSI reduction effort, there was a need to re-design and align various representative elements of the mesosystem to address challenges with variable standards, reliability in practice, and sustainability. This mesosystem, which we called the “CLABSI governance team,” would also serve as the “glue” connecting the microsystems and the microsystems with the macrosystem (enterprise leadership).

We describe a systems-based A3 approach to implement standards across all microsystems with adaptations allowed for specific situations approved through the CLABSI governance team (mesosystem) and an overarching macrosystem involvement creating a process for accountability at all levels.

## METHODS

**Fig. 1.**
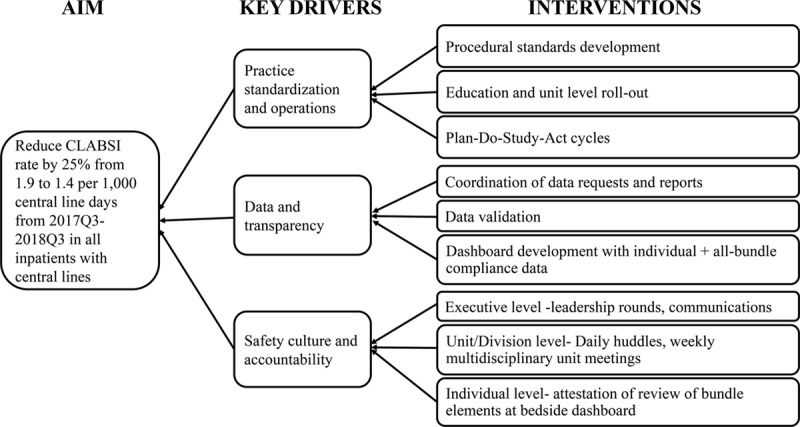
CLABSI A3 showing the main buckets of key drivers and abridged version of the interventions. Q indicates quarter.

We conducted this improvement initiative at a 395-bed academic, university-affiliated, freestanding children’s hospital. The initiative was deemed a quality improvement project and not human subjects research. Therefore, review and approval by the institutional review board were not required. We used Lean and Model for Improvement methodologies in our quality improvement initiative.^14^ The multidisciplinary CLABSI governance team (the mesosystem) included leadership from quality, nursing and shared governance, and infection prevention and control. The team developed a systems-based A3 with key drivers and countermeasures targeted toward adherence to the complete bundle. A3 is a problem-solving tool that involves defining the problem, articulating an achievable goal, analyzing causes, identifying key drivers and specific countermeasures, and making iterations based on results. The key drivers included (1) practice standardization and operations, (2) data and transparency, and (3) safety culture and accountability (Fig. 1 and Supplemental Digital Content at http://links.lww.com/PQ9/A172 for Figure). We identified these key drivers through lessons learned from several years of CLABSI reduction work and with input from unit-level physician and nurse leaders. Implementation of the A3 involved additional members from information systems, analytics, vascular access, interventional radiology, physician and nurse leaders, and front-line providers.

For work on key driver “practice standardization and operations,” nursing utilized the Ferrari Method for Practice Standardization, which marries lean methodology and evidence-based practice to address procedural practice standardization, in this case, central line care.^15^ Teams of front-line nurses from all pertinent units and relevant stakeholders such as quality and infection prevention and control representatives convened to create agreed-upon standards for central line care and device selection. The team evaluated the current state and developed evidence-based interventions targeted at CLABSI reduction. This nursing-led team met over 2 days to develop standardized approaches relevant to central line maintenance practices (changing of needleless connector, changing of intravenous tubing, chlorhexidine bathing, scrubbing the hub, protection of central line environment, procedure for blood culture drawing) and implementation of bundle round audit process (which entails bedside rounding on all elements of the bundle for every central line).

The key driver, “data and transparency,” focused on leveraging existing patient-level data and developing unit-level data to inform individuals and unit leaders, respectively, on bundle compliance. Patient-level data focuses on ensuring that bedside nurses are reviewing bundle elements each shift as part of their daily workflow and that patient-level bundle adherence was reviewed at shift handoffs to ensure ongoing visibility. The unit-level data allow unit-level leadership to track adherence to the individual elements as well as all of the bundle elements. Through this system-wide unit level dashboard, we changed from capturing missing elements on a sample of patients to visibility on adherence with all elements for all patients at a unit and hospital-level. The clarity on standards and transparency of data allowed for identifying low adherence to specific bundle elements at a unit or hospital level, thereby prompting leadership to identify and resolve the barrier.

The third key driver, “safety culture and accountability,” focused on creating a culture of accountability both in general and specifically on the importance of bundle adherence in CLABSI prevention. The approach incorporated general elements of “just culture” of individual accountability with improvement in systems.^16^ There were several interventions instituted specifically for the CLABSI reduction effort. First, we used the patient-level dashboard to ensure that bedside nurses were aware and accountable for bundle elements for the patients in their care. Second, we used the unit-level dashboard to ensure unit leaders were engaged and accountable for the performance of their unit on adherence to the all-or-none central-line bundle. Third, the quality leadership team implemented a cadence of rounding in the units. Finally, our executive leadership communicated consistent messaging to front-line staff and faculty about expectations and the importance of bundle adherence and hand hygiene.

We identified CLABSI using standardized surveillance definitions used by the Centers for Disease Control and Prevention National Healthcare Safety Network.^17^ We report quarterly hospital-wide CLABSI rates defined as the total number of CLABSI events per 1,000 central line days per quarter. We also report our hospital’s quarterly SIR for CLABSI, which reflects the observed-to-expected ratio of infections adjusting for hospital characteristics. CLABSI SIR >1.0 indicates that there were more CLABSIs observed than predicted; conversely, a SIR <1.0 indicates that there were fewer CLABSIs observed than predicted.^18^ We used statistical process control (SPC) charts to display the CLABSI rate and SIR over time. The center-line represents the average with 3 standard deviations upper and lower control limits included in the chart. The control charts were produced using the plug-in QI Macros for Excel. While both CLABSI rate and SIR exclude mucosal barrier injury-related bloodstream infection, SIR adjusts for the facility and/or patient-level factors that contribute to HAI risk. Data included for analysis were from January 1, 2015, to June 30, 2019. For comparison, we also examined our hospital’s quarterly catheter-associated urinary tract infection (CAUTI) rates per 1,000 urinary catheter days during the same period.

To evaluate the impact of this multi-faceted intervention on CLABSI rates, we used a Poisson regression model to measure the effect of the intervention on quarterly changes in CLABSI rates, controlling for baseline trends. Since the intervention incorporated several key drivers over several quarters, we considered the intervention period to extend from October 2017 to June 2018, and we excluded that period from the analysis. Thus, our pre-intervention period ranged from January 2015 to September 2017, and our post-intervention period included data from July 2018 to June 2019. Our independent variables included the intervention (post versus pre-intervention), time (secular trends based on quarterly data), and an interaction term to determine whether the intervention resulted in a change in slope for the comparison of the post versus pre-implementation period. Since the interaction term was not significant, we present the most parsimonious CLABSI model here. We present incidence rate ratios with 95% CI. A *P*-value of < 0.05 was considered statistically significant.

## RESULTS

We implemented our multifaceted intervention beginning in the fourth quarter of 2017 with a focus on practice standardization. Simultaneously, we developed and tested our patient-level and unit-level dashboards based on the practice standards developed. Our data and transparency and accountability teams began rollout and completed implementation in all units by the third quarter of 2018.

### Implementation of CLABSI A3—NICU Microsystem Example

Historically the NICU made modifications to enterprise-wide initiatives because of the specific needs of their neonatal population, which resulted in the evolution of their practices away from our expected standard. Units governed their practices, and alignment with organizational standards or teams was not prioritized. In this initiative, our NICU team realigned efforts within the context of a mesosystem focused on CLABSI prevention, with specific, justifiable requests for adaptation of the standards for their population. For example, the NICU team specifically reviewed the evidence for the safety and effectiveness of chlorhexidine bathing in the NICU population. After their review, they requested the use of a bathing protocol that specifically incorporated the use of specific products and frequency by gestational age, which the CLABSI governance team approved based on their evidence review. Also, the NICU works in an open bay environment, where there were concerns about the role of the physical environment and risk for infections. Thus, the NICU also emphasized the importance of adherence to unit-specific environmental hygiene practices as a key component of their infection prevention efforts. Despite these unit-specific adaptations, the overall emphasis remained on all-or-none bundle adherence and accountability for practice. Data on bundle element adherence were provided to bedside nurses and unit leadership. Unit leadership conducted bedside bundle rounds to provide just-in-time education to bedside staff on best practices for individual bundle elements to ensure consistency and high reliability. Hospital leadership conducted rounds with the NICU teams to emphasize the importance of CLABSI prevention to the organization and to further enhance the sense of accountability at all levels.

### Impact of Intervention on CLABSI rates

**Fig. 2.**
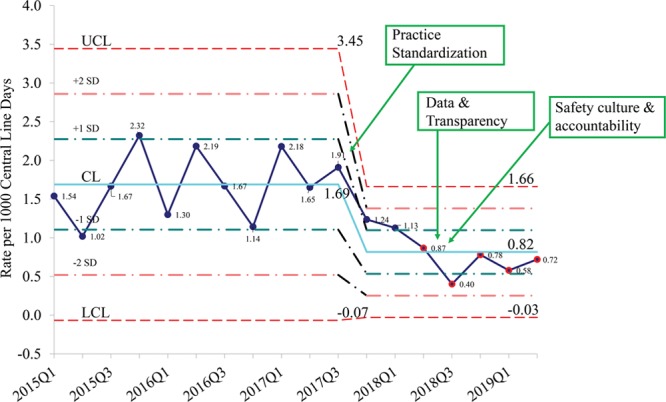
SPC chart with quarterly CLABSI rate per 1,000 central line days for the years 2015–2019 with time-line of interventions for the key drivers. Q indicates quarter; UCL, upper control limit; CL, centerline; LCL, lower control limit.

Figure 2 shows the quarterly CLABSI rates from January 2015 to June 2019, annotated with the implementation of interventions for the key drivers. Baseline quarterly CLABSI rates during the pre-intervention period ranged from 1.0 to 2.3 CLABSIs per 1,000 central line-days. During the post-intervention period, the quarterly CLABSI rate ranged from 0.4 to 0.7 per 1,000 central line days. When adjusting for secular trends, we found a statistically significant decrease in the post versus pre-intervention CLABSI rate of 71% (Table 1).

**Table 1.**
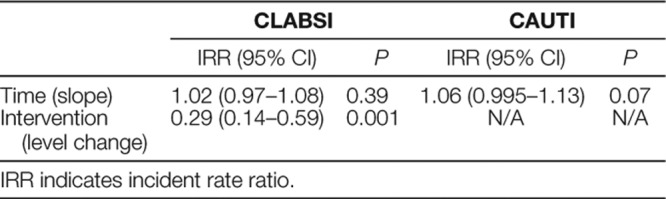
Changes in Quarterly Rates Over Time for CLABSI and CAUTI

**Fig. 3.**
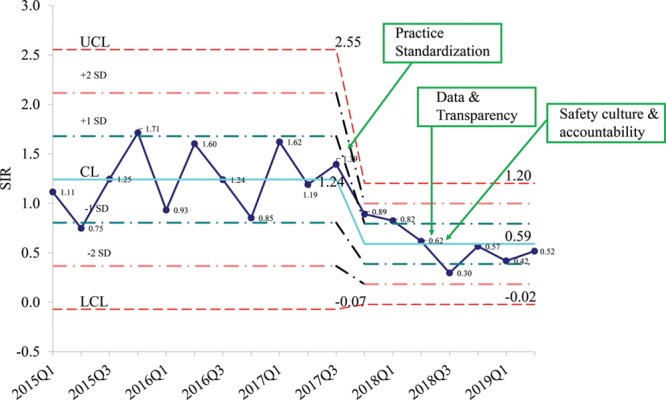
SPC chart with CLABSI quarterly SIR 2015–2019Q2. Q indicates quarter; UCL, upper control limit; CL, centerline; LCL, lower control limit.

Similarly, we observed a significant decrease in the CLABSI SIR in the post-intervention period compared with the pre-intervention period (Fig. 3). Based on the control limits in the pre-intervention period, the trend of 5 consecutive points for the CLABSI rate and SIR below 1 SD of the center-line in the post-period indicate a process shift in the mean (Figs. 2 and 3). Even with the exclusion of the NICU (which had a significant reduction in rate), there was an overall decline in the hospital-wide CLABSI rate (Supplemental Digital Content at http://links.lww.com/PQ9/A173 for Table).

## DISCUSSION

There was a decline in the CLABSI rate and SIR following a baseline of sustained high rates, despite several previous interventions over many years. The strong microsystem culture in our healthcare system remained critical to this success. We identified variability in standards across microsystems as an important contributor to the challenges in the previous CLABSI reduction efforts. The formation of the mesosystem to interface with the microsystems, develop standards, coordinate resource utilization, and serve as the “glue” between the microsystems as well as providing a reporting structure and leveraging the vision and engagement of the macrosystem was crucial in this improvement initiative.^11,19^ To our knowledge, this is the first reported experience identifying the important role of the mesosystem in CLABSI reduction at an institution.

One of the limitations of our project includes the absence of a control group. However, in comparing with another HAI such as CAUTI in the same period, where we did not have a similar mesosystem-focused initiative, we did not observe declines. Second, our findings may have been due to a Hawthorne effect (changed behaviors while being observed), since there was consistent and very visible macrosystem focus on the goal of CLABSI reduction. Yet, a similar macrosystem focus in several years prior did not yield similar reductions in CLABSI. Finally, our post-intervention period only included four quarters of data; thus, we will need to continue monitoring to ensure the sustainability of the mesosystem intervention. However, by not adding to the workload of our front-line providers and streamlining it through a clear process, we believe the results of this intervention are sustainable. Ongoing work to integrate other best practices such as reducing placement of lines, prompt removal of lines, and reducing central line access will potentially further reduce the incidence of CLABSIs.

While the bundle itself is incredibly important, creating a system to allow for adherence to *all* of the bundle will have to be tailored to the challenges that could be unique to a healthcare system. In this initiative, we used the existent robust microsystems and re-designed a mesosystem to create and implement the improvement tool. We are using our experience with CLABSI to address reduction efforts of other healthcare-associated conditions, thereby achieving the goal of standardization.

## ACKNOWLEDGMENTS

Assistance with the initiative: We acknowledge the following members of Stanford Children’s Health and Lucile Packard Children’s Hospital for their assistance - Jane Russell from Nursing leadership; Shabnam Gaskari from Pharmacy leadership; Heidi Chan from Quality Improvement; Derek Garnholz and Margaret Godin from Information Systems; Ling Loh from Analytics; Stephanie Klee from Interventional Radiology; and Andrew Ward, Augustine Chemparathy, Ron Li, Simran S. Mirchandani, Martin Seneviratne, Andrew Shin, and David Scheinker from the Systems Utilization for Stanford Medicine (SURF).

## DISCLOSURE

The authors have no financial interest to declare in relation to the content of this article.

## Supplementary Material


