# Supplementary material for: Reduction of Central Line-associated Bloodstream Infection Through Focus on the Mesosystem: Standardization, Data, and Accountability
Source: Pediatr Qual Saf. 2020 Mar 25;5(2):e272. doi: 10.1097/pq9.0000000000000272 (PMC7190265; doi:10.1097/pq9.0000000000000272)
Supplement: Supplementary file 1 [file pqs-5-e272-s001.pdf]

SDC, Figure. Expanded A3

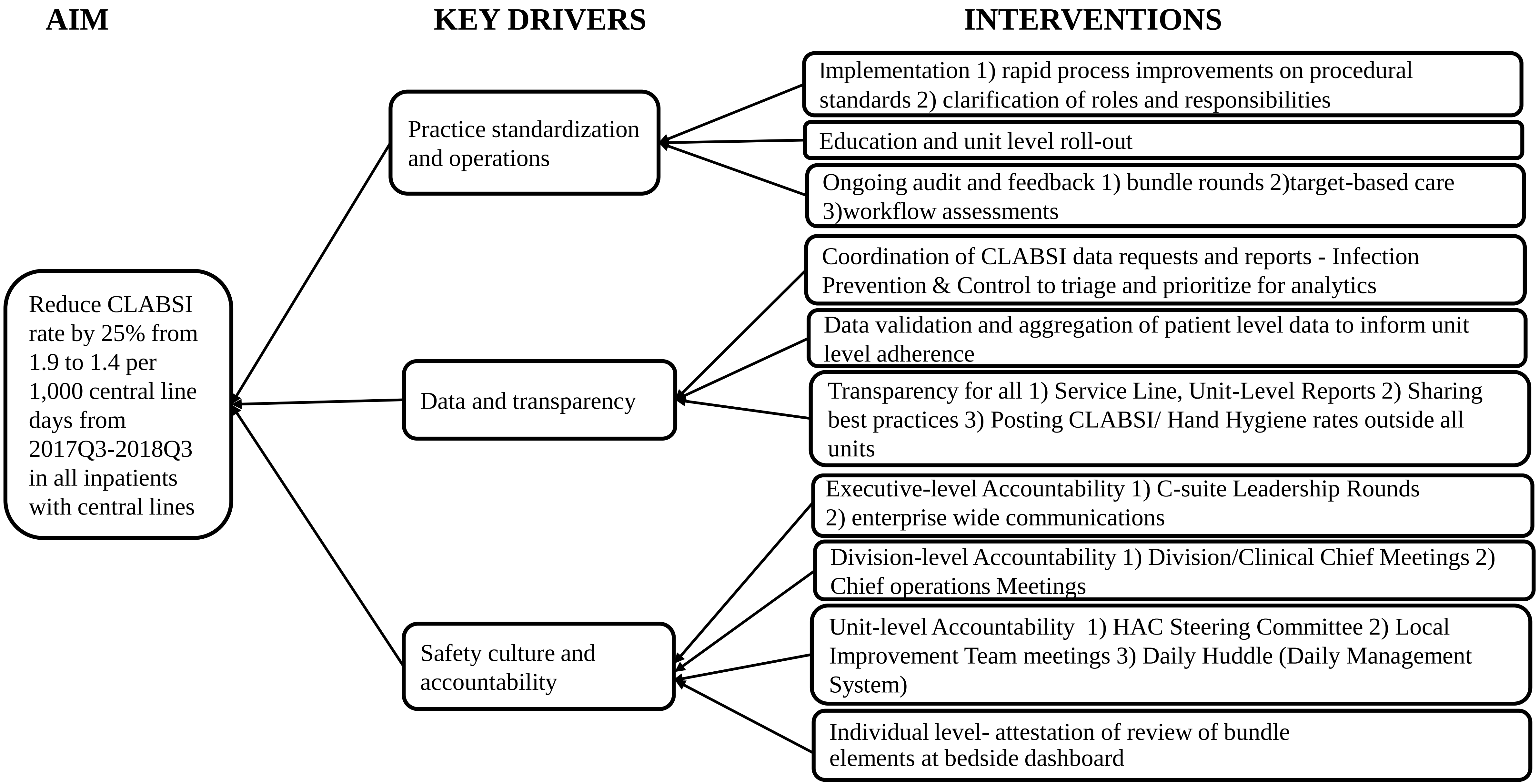

CLABSI, central line-associated bloodstream infection; Q, quarter; HAC, healthcare-associated conditions
