# Supplementary material for: Reduction of Central Line-associated Bloodstream Infection Through Focus on the Mesosystem: Standardization, Data, and Accountability
Source: Pediatr Qual Saf. 2020 Mar 25;5(2):e272. doi: 10.1097/pq9.0000000000000272 (PMC7190265; doi:10.1097/pq9.0000000000000272)
Supplement: Supplementary file 2 [file pqs-5-e272-s002.pdf]

SDC, Table. Hospital-wide and neonatal intensive care unit central line-associated bloodstream infection rate during years 2015-2018

Year	Hospital-wide CLABSI rate	NICU CLABSI rate	Hospital-wide CLABSI rate without NICU
**2015**	1.64	2.46	1.46
**2016**	1.59	2.32	1.45
**2017**	1.74	2.29	1.65
**2018**	0.79	0.27	0.86

CLABSI, central line-associated bloodstream infection; NICU, neonatal intensive care unit
